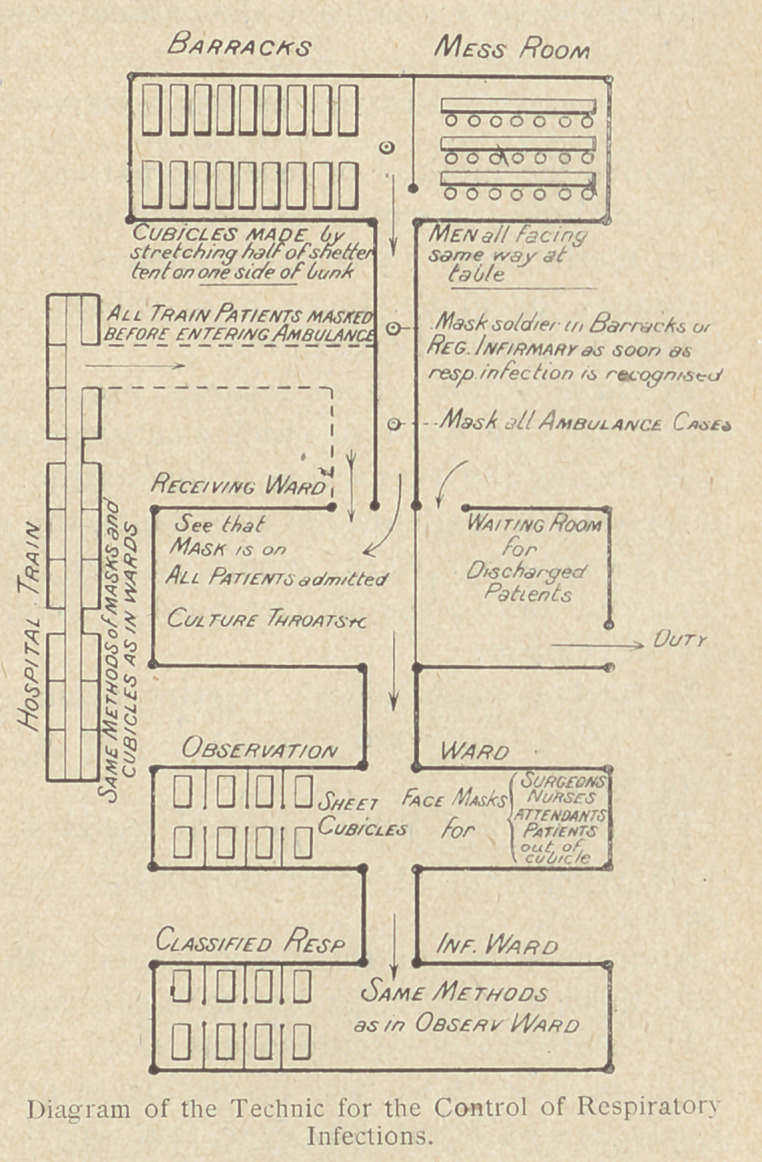# The Limitation and Control of Streptococcus and Other Respiratory Infections

**Published:** 1918-11

**Authors:** Joseph A. Capps


**The Limitation and Control of Streptococcus and other Respiratory Infections.** Lt.-Colonel Joseph A. Capps, M. C.

I propose to relate briefly our experience with streptococcus infections during the last year at Camp Grant, and to dwell more at length on the measures employed in combating the streptococcus and other respiratory diseases.

In the fall of the year we were concerned chiefly with the classical lobar pneumonias, with pneumococci in the sputum and with a normal clinical course. The few pneumococcus empyemas that occurred were of a mild type.

The first epidemic of streptococcus infections began in December, 1917, with the arrival of 6500 troops from Jefferson and Columbus Barracks, These soldiers came into camp with colds and rapidly filled the wards with cases of rhinitis, purulent bronchitis, broncho-pneumonia and empyema. Some of the broncho-pneumonias were primary streptococcus infections, and showed streptococci in the empyema pus. There were also cases of primary lobar pneumonia with pneumococci in the sputum, many of whom developed a streptococcus empyema.

A second smaller outbreak of streptococcus infection of a different nature occurred in the last week of January. The onset was explosive and characterized by intense sore throat, usually with a fibrinous exudate, by marked swelling of the cervical lymphatics, by fever and extreme prostration. Suppuration of the glands devel.

oped in several cases, but neither bronchitis nor pneumonia. Throat cultures showed streptococci and no diphtheria bacilli. The clinical picture suggested the possibility of a milk borne epidemic and further investigation revealed very strong evidence to support this explanation of its source. Pasteurization of the milk was begun and these cases gradually disappeared.

A third wave of streptococcus infection appeared in May, when a large number of negro troops from North Carolina entered the Division. Clinically the cases were like those in the first epidemic, entering the hospital for rhinitis and bronchitis, and frequently developing broncho-pneumonia and empyema.

It was a fact often commented upon that the mortality was always greater at the beginning of a fresh outbreak than later, as if the virulence became attenuated.

In over 900 cases of measles only 20 were complicated with broncho-pneumonia, most of them considered to be of streptococcal origin.

When we compare the pathogenic organisms responsible for the various respiratory infections in our home camps, the streptococcus easily takes the lead, both in causing the greatest number of seriously ill and the greatest number of deaths. As a primary infection the streptococcus has a formidable record, but as a secondary infection, especially in pneumonia and measles, the streptococcus has been more dangerous than all other organisms together.

### Preventive Measures.

Our measures for combating streptococcus infections, however, are not essentially different from those indicated in other infections of the respiratory tract.

All the respiratory infections, including streptococcus, influenza, pneumonia, measles, meningitis, whooping cough, scarlet fever, and mumps are transmitted by fine droplets of germ-laden mucus, conveyed by means of coughing, sneezing, and talking, from one person to another.

The danger of transmission from one individual to another varies directly with the distance separating them. The closer the proximity, the greater likelihood of spreading the contagion. It is to be expected, therefore, that the close proximity of soldiers in barracks, in receiving wards, and in hospital wards, should tremendously favor the spread of these diseases.

Every effort should be made to avoid overcrowding, but this can only be mitigated, not prevented.

Having soldiers in barracks sleep so that the head of one is opposite the feet of his neighbor is a helpful measure. The half of a shelter tent stretched between adjoining bunks is even more effective.

In wards a system of cubicles has met with general approval. These are formed by sheets suspended from wires, or from the mosquito netting frames, which are a part of our standard beds.

From the time that the base hospital at Camp Grant was opened, face masks of gauze have been worn by physicians, nurses and ward men in the contagious wards. This practice was placed on a sound clinical and experimental basis at the Durand Hospital for Infectious Diseases by Weaver (*Journal of A. M. A.*, Jan. 12, 1918), who for more than a year previous had by this method protected his staff from diphtheria and scarlet fever; he also proved by repeated throat cultures that this method greatly reduced the incidence of diphtheria carriers among the nurses.

Diagram of the Technic for the Control of Respiratory Infections.

The use of the gauze face masks in this way at Camp Grant and other hospitals has given similar protection to doctors and nurses. However, this does not give the patients protection one from another. So long as a patient remains isolated in the cubicle he is protected; when he leaves the cubicle he endangers others, and is himself exposed to cross infection. As a result of numerous cross infections, particularly of streptococcus, we began during the second week of February, 1918, (*Journal A. M. A.*, Mar. 30, 1918) the use of the face mask[1] on patients in all wards where respiratory

1. Major David A. Haller, former Chief of Laboratory at Camp Grant, has made an infections were treated. Each patient was issued daily a clean mask, which, when not in use, was pinned to the cubicle sheet.

The patients were told that the cubicle is like the dug-out in a gassed area; as long as one remains inside, the mask is unnecessary, but it is dangerous to leave the cubicle unmasked.

### Results of the masking system.

Before the method of masking patients was introduced, and the cubicle alone was employed, we had ten instances of cross infection with scarlet fever in wards occupied by other diseases. In four instances, or 40 o/o, there were subsequent cases of scarlet fever during the week of quarantine.

In three wards where measles broke out as a cross infection, there was one ward in which a subsequent case of measles developed during the two weeks of quarantine. After masks were used universally by patients and attendants, the results were as follows:

In twenty-four wards where scarlet fever appeared as a cross infection, there was only one ward or 4 o/o, in which a subsequent case developed.

In twelve wards where measles occurred as a cross infection, there were two wards, or 17 o/o, in which a subsequent case developed.

To summarize: Before general masking, in thirteen wards with cross infection, there were five wards, or 38 o/o, with subsequent cases.

After masking, in thirty-six wards with cross infection, there were three wards, or 8.3 o/o, with subsequent cases.

The statistics in streptococcus cross infections cannot be tabulated, because there was no period of quarantine after exposure. It may be significant, however, that only twenty cases of bronchopneumonia developed in over 900 cases of measles, although streptococcus infections were prevalent.

It soon became evident, however, that many patients were exposed to cross infection in the regimental infirmaries, in the crowded ambulances, and in the receiving ward. The following measures were adopted:

experimental study to determine the best type of mask. His report will soon appear in the *Journal of the A. M. A.* He advises the use of 4 layers of 24 × 20 or 28 × 24 gauze, or 3 layers of 32 × 26 gauze. Masks should be 8 inches long, with edges turned in and stitched. They should be 5 inches wide. A braid a yard long should be sewed along the upper and lower borders, so as to leave a free end 14 inches long at each side. The masks should be marked on the face side by a stitch of black thread.

At the regimental infirmary every case with respiratory infection was masked as soon as recognized.

In the ambulance every patient was masked, regardless of his ailment.

In the receiving ward every ambulatory patient who entered the hospital was masked at the door, and all patients continued to wear the mask until they reached their cubicles in the wards.

We all believed that these precautions definitely reduced cross infections.

### Summary.

(A) Streptococcus infections are among the most formidable of the respiratory group. Epidemics of unmixed primary infection are severe, but the streptococcus infections, associated with pneumonia, measles, gas poisoning and probably with influenza, are more dangerous.

(B) All the respiratory infections, including the streptococcus and influenza groups, are transmitted by the germ laden droplets of mucus expelled from the mouth or nose in the act of coughing, sneezing or talking.

(C) To limit the spread of these infections in the overseas service, the following measures are advocated.

(1) In *barracks* avoid overcrowding. Separate patients at night by stretching the half of a shelter tent on the side of each bunk. As soon as a case of febrile respiratory infection is recognized, he should be masked by the regimental surgeon and sent to the hospital.

So arrange mess tables that all soldiers face the same direction while eating.

(2) Mask all *ambulance* patients.

(3) On *hospital trains* hang a sheet lengthwise over the front half of each tier of 3 cots. Mask all surgeons, nurses and personnel while on duty with patients. Sitting patients with cough should be masked, and any bed patients who leave their cubicles. Mask all patients before removal into ambulances.

(5) In the *receiving ward*, station an orderly with a box of masks, with instructions to see that all patients admitted are masked. The masks should be worn until they have reached the shelter of their ward cubicles. Discharged patients should be kept separate from those awaiting admission.

(4) In all hospital wards where respiratory infections are treated, use the sheet cubicle. The face mask, made of 4 layers of gauze of good quality, should be worn by physicians, nurses and ward men when on duty in the wards, and by all patients when out of their cubicles. The patient should wear the mask when out of his cubicle at all times, except when in the wash room, where only one should enter at a time. Meals should be eaten in the cubicle. Smoking should be prohibited for these patients as it requires removal of the face mask and is harmful to the inflamed air passages.

(5) All eating ustensils should be sterilized after each meal.

(6) Masks should be disinfected by soaking an hour in 2 o/o cresol solution, then by boiling half an hour in soap and water.

(7) Finally, the time has come for the internist to take the same attitude toward respiratory infections that the surgeon has toward wound infections, and to develop a comprehensive technic for their limitation and prevention.

## Figures and Tables

**Figure f1:**